# Personalized Microbiome Modulation to Improve Clinical Outcomes in Pediatric Inflammatory Bowel Disease: A Multi-Omics and Interventional Approach

**DOI:** 10.3390/microorganisms13051047

**Published:** 2025-04-30

**Authors:** Adrian Goldiș, Radu Dragomir, Marina Adriana Mercioni, Christian Goldiș, Diana Sirca, Ileana Enătescu, Laura Olariu, Oana Belei

**Affiliations:** 1 Department of Gastroenterology and Hepatology, “Victor Babeș” University of Medicine and Pharmacy, 300041 Timișoara, Romania; goldis.eugen@umft.ro; 2 Department of Obstetrics and Gynecology, “Victor Babeș” University of Medicine and Pharmacy, 300041 Timișoara, Romania; 3 Faculty of Medicine, “Victor Babeș” University of Medicine and Pharmacy, 300041 Timișoara, Romania; marina.mercioni@student.umft.ro (M.A.M.); christian.goldis@student.umft.ro (C.G.); diana-cristiana.sirca@student.umft.ro (D.S.); 4 Applied Electronics Department, Faculty of Electronics, Telecommunications and Informatio Technologies, Politehnica University Timișoara, 300223 Timișoara, Romania; 5 Twelfth Department, Neonatology Clinic, “Victor Babes” University of Medicine and Pharmacy, 300041 Timișoara, Romania; enatescu.ileana@umft.ro; 6 First Pediatric Clinic, “Victor Babeș” University of Medicine and Pharmacy, 300041 Timișoara, Romania; olariu.laura@umft.ro (L.O.); belei.oana@umft.ro (O.B.); 7 First Pediatric Clinic, Disturbances of Growth and Development on Children Research Center, “Victor Babeș” University of Medicine and Pharmacy, 300041 Timișoara, Romania

**Keywords:** pediatric inflammatory bowel disease (IBD), gut microbiome, dysbiosis, microbiome-targeted therapy, microbiome modulation, dietary intervention, probiotic supplementation

## Abstract

Inflammatory bowel disease (IBD) is a complex disorder influenced by genetic, environmental, and microbial factors, with emerging evidence highlighting the gut microbiome’s role in disease pathogenesis. This study investigates the impact of microbiome-targeted interventions in pediatric IBD by integrating multi-omics analysis, including metagenomics, metabolomics, transcriptomics, and clinical biomarkers, to identify microbial dysbiosis patterns and potential therapeutic targets. A cohort of pediatric IBD patients underwent a personalized intervention involving dietary modifications, probiotic supplementation, and selective antibiotic therapy. Microbiome composition, inflammatory markers (fecal calprotectin, CRP), and disease activity scores (PCDAI/PUCAI) were assessed before and after treatment. At the 3-month follow-up, patients showed significant clinical improvement, with reduced stool frequency (*p* = 0.004) and improved stool consistency (*p* < 0.001). Symptoms such as bloating and abdominal pain decreased, while energy levels increased (*p* < 0.001). Dietary changes included higher fruit, meat, and dairy intake, and lower fast-food and sweets consumption (*p* < 0.001). Physician assessments classified 90% as “improved”, reinforcing the effectiveness of personalized microbiome interventions. Microbiome-targeted interventions (diet, probiotics, and selective antibiotics) improved pediatric IBD outcomes by reducing pathogenic bacteria and increasing short-chain fatty acid (SCFA)-producing species, lowering inflammation and symptoms. Early-life factors (cesarean birth, and formula feeding) influence IBD risk. Personalized diets enhanced microbial balance. Integrating multi-omics supports precision medicine, offering microbiome-based biomarkers and reducing immunosuppressive reliance.

## 1. Introduction

Inflammatory bowel disease (IBD) is a chronic condition marked by recurrent episodes of inflammation in the gastrointestinal tract. It encompasses two primary subtypes: Crohn’s disease (CD) and ulcerative colitis (UC) [1]. In recent decades, the incidence of pediatric-onset IBD has risen significantly worldwide, particularly in industrialized regions [2]. This increase highlights the intricate relationship between genetic susceptibility and environmental influences in disease pathogenesis [3]. Although IBD was historically considered a disorder of adulthood, recent epidemiological findings indicate that up to 25% of cases manifest in childhood or adolescence [4]. Pediatric IBD often presents with a more aggressive disease course, characterized by extensive intestinal involvement, increased corticosteroid dependency, and a greater need for early therapeutic intervention compared to adult-onset cases [5]. Despite advancements in understanding IBD pathophysiology, its precise etiology remains unclear, particularly in pediatric populations where unique developmental, immunological, and environmental factors may significantly influence disease onset and progression [6].

One of the most critical factors in IBD pathogenesis is the gut microbiome, a dynamic and diverse community of microorganisms in the intestines [7]. This ecosystem, composed of bacteria, viruses, fungi, and archaea, is vital in immune regulation, epithelial barrier maintenance, and metabolic function. In healthy individuals, a balanced microbiome supports mucosal immunity and prevents excessive inflammation. However, IBD is associated with significant microbial alterations, known as dysbiosis, which include reduced microbial diversity, expansion of pro-inflammatory taxa (e.g., Enterobacteriaceae), and depletion of beneficial commensals. These changes contribute to epithelial barrier dysfunction, increased intestinal permeability, and chronic mucosal inflammation—all hallmarks of IBD pathology [8]. While dysbiosis is observed in adult and pediatric IBD, early-life environmental factors may impact microbial development, influencing disease susceptibility [9].

Environmental factors are crucial in shaping the gut microbiome and modulating immune responses, especially during early developmental windows [10]. Perinatal factors, such as mode of delivery, breastfeeding, antibiotic exposure, and maternal diet, influence microbial colonization in infancy and may predispose individuals to immune dysregulation and inflammatory diseases later in life [11]. Dietary habits are another key determinant of microbiome composition. Westernized diets, characterized by high intake of ultra-processed foods, refined sugars, and saturated fats, are strongly linked to reduced microbial diversity and increased intestinal inflammation [12,13]. Conversely, fiber-rich diets, abundant in prebiotics and polyphenols, promote microbial resilience and exert anti-inflammatory effects that may protect against IBD [12]. Given that pediatric IBD is often diagnosed during a critical period of immune and microbial maturation, understanding the impact of early-life exposures is essential for identifying preventive and therapeutic strategies [10,11].

Although genetics contribute to IBD, they do not fully explain its rising incidence, shifting research toward the microbiome’s role in the disease [14]. Strategies like FMT, probiotics, and dietary interventions show promise in restoring microbial balance, but their efficacy and mechanisms require further validation through longitudinal studies [15].

Current microbiome research on IBD is limited by a fragmented approach, often analyzing microbial composition or function without integration. Cross-sectional designs further hinder causal insights. Advances in multi-omics now enable a deeper understanding of host–microbiome interactions, making integrated analysis crucial for developing personalized treatments [16].

This study goes beyond microbiome characterization and aims to directly modulate the gut microbiota to improve clinical outcomes in pediatric IBD. By integrating metagenomics, blood data, advanced disease profiling questionnaires, and imaging findings, we identify individualized inflammatory microbiome signatures that serve as diagnostic and therapeutic targets. Our intervention focuses on personalized microbiome modulation by implementing targeted dietary modifications to restore short-chain fatty acid (SCFA)-producing species (e.g., *Faecalibacterium prausnitzii*, *Akkermansia muciniphila*), probiotic and prebiotic supplementation tailored to individual microbial deficits, and strategies to reduce pathogenic overgrowth (e.g., *Bacteroides fragilis*, *Clostridium difficile*, adherent-invasive *Escherichia coli*) through a combination of dietary adjustments and microbiome-targeted interventions.

By integrating longitudinal clinical monitoring with microbiome reprogramming, we assess the direct impact of these interventions on gut inflammation, disease activity, and patient-reported outcomes. This study thus shifts the paradigm from merely associating dysbiosis with disease to actively intervening in a precision medicine approach. Our findings aim to pave the way for personalized microbiome therapeutics, reducing reliance on systemic immunosuppression and offering a new frontier in pediatric IBD management.

## 2. Materials and Methods

### 2.1. Study Design and Population

This study employed a longitudinal interventional design to assess the effects of personalized microbiome modulation on clinical outcomes in pediatric IBD patients. A total of 30 pediatric patients (20 with CD, 10 with UC), aged 4–18, were recruited from a tertiary care center.

The inclusion criteria were as follows:

- Age range: Participants aged 4–18 years.
- Confirmed IBD diagnosis: Diagnosis of CD or UC based on clinical, endoscopic, and histopathological assessment [17].
- Disease activity: Patients with active or remitting IBD are monitored via the pediatric CD activity index (PCDAI) [18] or the pediatric UC activity index (PUCAI) [19].
- Stable treatment regimen: Patients must have been on stable medical therapy (e.g., immunomodulators, biologics) for at least 3 months before enrollment.
- Ability to adhere to intervention protocol: Willingness to follow the personalized microbiome modulation protocol, including dietary modifications, probiotic/prebiotic supplementation, and regular clinical follow-ups.
- Written informed consent: Signed informed consent from legal guardians and assent from patients where applicable.

The exclusion criteria were as follows:

- Recent antibiotic use: Patients who used antibiotics within 3 months before enrollment, as this could significantly alter microbiome composition.
- Active infections: Presence of acute infections (bacterial, viral, fungal, or parasitic) at the time of recruitment.
- Concurrent autoimmune disorders: Patients with other diagnosed autoimmune conditions (e.g., celiac disease, autoimmune hepatitis, systemic lupus erythematosus).
- Severe comorbidities: Presence of chronic metabolic, neurological, or genetic disorders that may interfere with microbiome analysis or dietary interventions.
- Severe malnutrition: Patients with severe malnutrition (BMI < 3rd percentile for age and sex) requiring parenteral nutrition.
- Non-compliance: Inability to adhere to the study protocol, including refusal to comply with dietary changes, probiotic supplementation, or follow-up visits.
- Prior fecal microbiota transplant (FMT): Patients who have previously received FMT, as this could confound microbiome analysis.
- Pregnancy or lactation: Female pregnant or breastfeeding participants at the time of recruitment.

**Figure 1:**
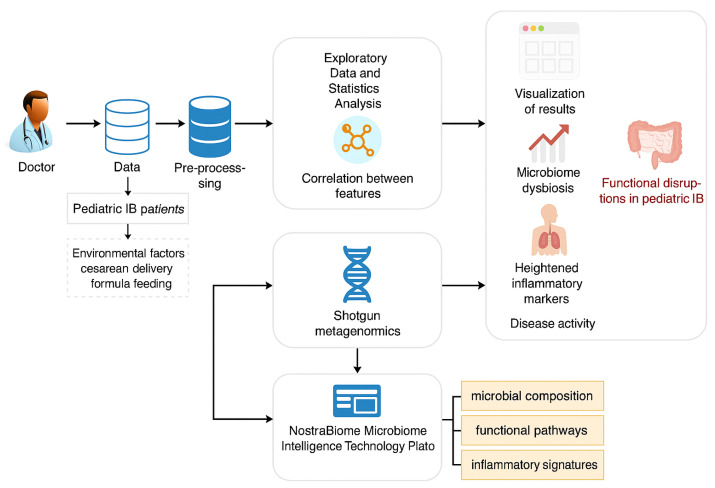
Workflow for integrating clinical data, environmental factors, and microbiome analysis in pediatric IBD.

Figure 1 illustrates a workflow for studying pediatric IBD using clinical data, environmental factors, and advanced microbiome analysis technologies. Doctors collect data on pediatric IBD patients, including patient details and ecological factors like cesarean delivery and formula feeding. After preprocessing the data, exploratory statistical analysis identifies correlations between microbiome dysbiosis, heightened inflammatory markers, and disease activity.

### 2.2. Intervention Protocol

Patients followed a three-month personalized microbiome modulation protocol, which included dietary modifications to promote short-chain fatty acid-producing bacteria (*Faecalibacterium prausnitzii*, and *Akkermansia muciniphila*) while reducing pro-inflammatory taxa, probiotic and prebiotic supplementation tailored to individual microbial deficiencies, and targeted dietary interventions to limit the overgrowth of pathogenic species (*Bacteroides fragilis*, *Clostridium difficile*, and *E. coli*); nutritional recommendations were adjusted based on baseline microbiome analysis and further modified according to microbial shifts and clinical responses. Baseline dietary habits were evaluated through a standardized semi-quantitative food frequency questionnaire (FFQ) completed by patients or caregivers during enrollment. The selected food categories were chosen based on their known impact on gut microbiota composition, inflammatory potential, and relevance to IBD dietary management. For patients with suspected lactose intolerance or dysbiosis involving lactose-fermenting species, lactose-free dairy alternatives were recommended during the intervention.

Interventions were individualized based on each patient’s baseline microbiome profile. For instance, patients with overrepresentation of *Clostridium difficile*, *Bacteroides fragilis*, or *Escherichia coli* were recommended targeted antibiotics (e.g., Rifaximin, Metronidazole), while those with low levels of SCFA-producing taxa (*Faecalibacterium prausnitzii*, and *Akkermansia muciniphila*) received enhanced prebiotic and fiber-rich dietary recommendations. Probiotic supplementation was customized, with specific strains selected according to detected microbial deficits. Microbiome reports and functional pathway analyses (e.g., SCFA biosynthesis, oxidative stress) informed these adjustments. For example, a patient with high *Bacteroides fragilis* and depleted *Faecalibacterium* received both Metronidazole and dietary adjustments to boost butyrate production.

Environmental and dietary exposures were also recorded at baseline to provide context for microbiome interpretation. Data included frequency of fast-food and processed food consumption, intake of fruits and vegetables, use of unprocessed milk, and primary sources of drinking water (bottled vs. private well). These factors were considered in both the initial interpretation of dysbiosis and the design of personalized dietary recommendations. In patients with more severe microbial imbalance and environmental risk exposures, more intensive dietary adjustments and prebiotic strategies were implemented.

The intervention protocol included individualized dietary, probiotic, and—when indicated—antibiotic components applied over a 12-week period. Patients followed a low-FODMAP [20,21], fiber-rich diet tailored to tolerance and nutritional needs, with a recommended daily fiber intake of 25–30 g. Probiotics (e.g., *Lactobacillus rhamnosus GG*, *Saccharomyces boulardii*) were administered once daily in capsule form (10⁹ CFU), selected based on microbial deficiencies. Frequently used strains included *Saccharomyces boulardii*, *Lactobacillus rhamnosus GG*, and *Bifidobacterium infantis*, among others. Doses ranged from 10⁹ to 10^10^ CFU/day, administered over the 12-week period. In some cases, multi-strain probiotic blends were recommended to restore microbial balance and enhance SCFA production. Strain selection and dosage were based on current ESPGHAN and WGO guidelines and adapted to pediatric needs and tolerance. In patients with documented overgrowth of pro-inflammatory taxa, targeted antibiotic courses (Rifaximin 200 mg TID or Metronidazole 250 mg BID) were prescribed for 7–10 days. All interventions were reviewed and adjusted at 3-month follow-up based on clinical and microbiological response.

A personalized dietary plan, following low-FODMAP principles, was developed and adjusted according to each patient’s clinical symptoms and microbial imbalance. Patients with high levels of potentially pro-inflammatory bacteria (e.g., *Clostridium bolteae*, *Bilophila wadsworthia*) were advised to eliminate fermentable carbohydrates and raw foods, while increasing cooked soluble fiber sources such as oats, bananas, carrots, and zucchini. Fermented foods (e.g., yogurt, kefir, sauerkraut) were recommended in cases with low microbial diversity. Dietary counseling was provided by a nutritionist at baseline and reinforced monthly.

Compliance with the dietary plan was assessed through weekly food diaries maintained by caregivers, which were reviewed during monthly follow-up consultations. Patients were evaluated for symptom changes, and dietary plans were modified accordingly. Adherence was also monitored using a structured questionnaire that assessed avoidance of restricted foods and incorporation of recommended ones. Adjustments were made in response to clinical evolution and, when available, follow-up microbiome analysis.

All patients and caregivers received standardized sleep hygiene advice to support overall health and gut–brain axis regulation. Recommendations included maintaining a consistent sleep–wake schedule, avoiding screen exposure at least one hour before bedtime, reducing evening intake of sugar or stimulants, and creating a calming bedtime environment. These measures were discussed during initial education and reinforced during monthly visits as sleep quality was considered a modifiable factor influencing intestinal inflammation.

### 2.3. Sample Collection and Multi-Omics Analysis

Fecal samples were collected at baseline for shotgun metagenomic sequencing and metabolomic profiling. In contrast, blood samples were analyzed for inflammatory biomarkers (fecal calprotectin, CRP, and IL-6) to assess correlations between microbial composition and disease activity. Serum levels of calcium, vitamin D, and iron were measured at baseline, and anemia screening was performed using complete blood count and ferritin levels. Multi-omics analyses were performed using the NostraBiome Microbiome Intelligence Technology Platform, integrating metagenomics to examine species composition and functional pathways, and metabolic predicted functions—based on strain quantity, frequency, and type—via our prediction platform, NostraBiome, to assess short-chain fatty acid levels and bile acid metabolism. At the three-month follow-up, clinical, dietary and microbiological parameters were re-evaluated using standardized questionnaires, laboratory analysis, and microbiome profiling in order to assess the impact of the intervention.

Patients were monitored through standardized clinical questionnaires, stool diaries, and validated dietary intake assessments. Disease activity was measured using the PCDAI and PUCAI.

### 2.4. Statistical Analysis

All statistical analyses were performed to evaluate the impact of personalized microbiome modulation on pediatric IBD patients. Descriptive statistics were used to summarize continuous variables, which were reported as mean ± standard deviation (SD), while categorical variables were expressed as frequencies and percentages.

Correlation analyses were conducted using Pearson’s or Spearman’s correlation coefficients, depending on data distribution, to explore relationships between dietary factors, environmental exposures, and clinical markers. These analyses helped identify statistically significant associations between dietary intake, inflammatory markers, and disease activity scores (PCDAI/PUCAI).

Chi-square or Fisher’s exact tests were used for group comparisons to assess associations between categorical variables, such as birth mode, probiotic consumption, and disease characteristics. Additionally, *t*-tests or Mann–Whitney U tests were likely employed to compare continuous variables between groups, such as CD and UC patients.

To assess the impact of the three-month intervention, paired *t*-tests or Wilcoxon signed-rank tests were used to compare pre- and post-intervention clinical and microbiome-related parameters. These tests evaluated significant changes in stool frequency, inflammatory markers, and dietary intake.

A significance threshold of *p* < 0.05 was applied, and multiple comparisons were adjusted where necessary. The statistical analyses ensured robust evaluation of microbiome modulation effects on clinical outcomes in pediatric IBD.

### 2.5. Ethical Considerations

This study followed the ethical principles outlined in the Declaration of Helsinki and adhered to national regulations for clinical research involving pediatric patients. Ethical approval was obtained from the Ethics Committee of the “Louis Țurcanu” Emergency Clinical Hospital for Children, Timișoara, under approval Nr—1693, dated 9 October 2024.

All participants and their legal guardians provided written informed consent before enrollment in this study. This study protected patient confidentiality, with all personal data anonymized and securely stored. Patients received standard care for IBD as per national guidelines, with the additional microbiome-targeted intervention being designed to complement existing treatments without introducing unnecessary risks.

The research team remained committed to minimizing potential risks and ensuring that all study interventions aligned with the best interests of pediatric patients. Participants had the right to withdraw from the study without affecting their standard medical care.

## 3. Results

### 3.1. Microbiome Modulation and Patient Characteristics

The microbiome modulation approach focused on correcting dysbiosis by addressing the overgrowth of pathogenic bacteria and supporting beneficial species (Table 1). Most patients exhibited an imbalance characterized by high levels of *Clostridium difficile*, *Bacteroides fragilis*, and *Escherichia coli*, along with a deficiency of *Akkermansia muciniphila* and *Faecalibacterium prausnitzii*. The predominant microbial composition included Firmicutes, Bacteroidetes, and Proteobacteria, with altered metabolic activity, such as reduced SCFA production and increased lactate levels, which are typically associated with gut inflammation.

To restore microbial balance, interventions included antibiotic therapy (Rifaximin, and Metronidazole) to reduce pathogenic bacteria, probiotics (*Saccharomyces boulardii*, *Lactobacillus rhamnosus,* and *Bifidobacterium longum*) to replenish beneficial microbes, and dietary modifications. Recommendations emphasized a low-FODMAP diet, increased fiber intake, prebiotics like inulin, and lifestyle changes such as improving sleep quality and maintaining oral hygiene.

The dataset includes pediatric IBD patients’ demographic, clinical, and dietary characteristics, offering insights into potential risk factors (Table 2). Most participants are adolescent males, with a high prevalence of cesarean births and short breastfeeding duration, factors that may influence microbiome development. The study cohort included 16 urban (53.33%) and 14 rural (46.66%) pediatric patients. Patients are evenly distributed between CD and UC, with symptoms typically including diarrhea, abdominal pain, and weight loss. Many consume bottled water and nearly half used probiotics before disease onset, while none reported taking nutritional supplements. Only 3 patients (10%) reported cessation of unprocessed milk consumption prior to disease onset, while the remaining 27 (90%) had no such history. Regular exposure to unverified water sources before disease onset was reported in 2 cases (6.66%), whereas 28 patients (93.33%) denied such exposure. For all demographic and clinical variables, 95% confidence intervals were calculated to reflect the precision of estimates, while *p* values were used to assess the statistical significance of group differences. These findings help identify correlations between microbiome composition, environmental exposures, and disease progression.

Based on the individual microbiome analyses, the most frequently reduced pathogenic taxa following intervention were *Clostridium difficile*, *Bacteroides fragilis*, *Escherichia coli*, *Bilophila wadsworthia*, and *Clostridium perfringens*. In parallel, key SCFA-producing species that increased included *Faecalibacterium prausnitzii*, *Roseburia intestinalis*, and *Akkermansia muciniphila*. These microbial shifts were consistent across patients and correlated with clinical improvement, including reduced inflammation and symptom severity.

**Figure 2:**
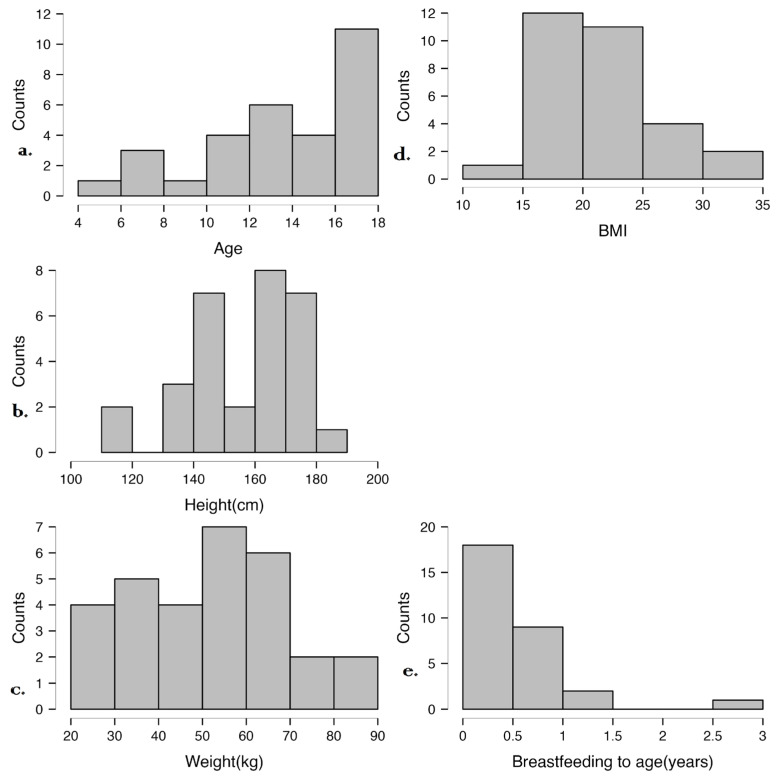
Distribution of anthropometric and early-life nutritional factors in the study cohort. (**a**) Age distribution, (**b**) Height distribution, (**c**) Weight distribution, (**d**) BMI distribution, (**e**) Breastfeeding duration.

Figure 2 illustrates the distribution of age, height, weight, BMI, and breastfeeding duration in the studied cohort. Most participants are adolescents with varied growth patterns, but their BMI values are within the normal range. Breastfeeding duration was generally short, which may influence microbiome development and immune maturation. These data provide context for analyzing associations between early-life factors, anthropometric measures, and disease characteristics.

**Figure 3:**
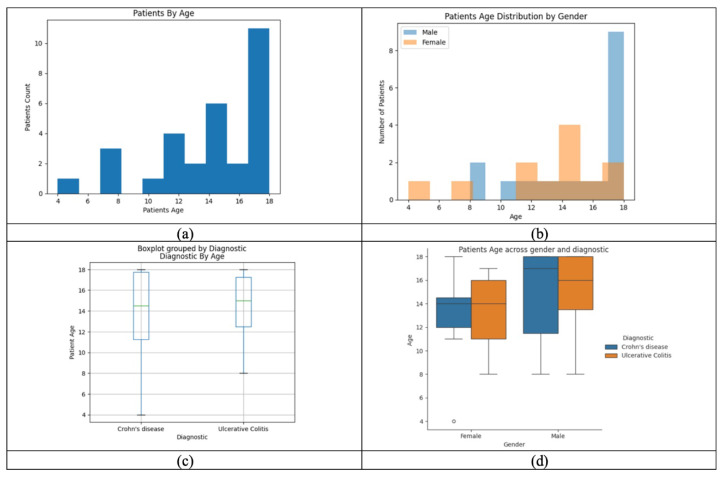
(**a**) Patients’ age distribution—most are aged 17–18. (**b**) Age by gender—older ages more common in males. (**c**) Age by diagnosis—similar distribution in Crohn’s and UC. (**d**) Age by gender and diagnosis—males with Crohn’s tend to be older.

Figure 3 analyzes the distribution of age, gender, and IBD subtype in the cohort, showing that most patients are adolescents. Gender and diagnosis patterns vary, with differences in age distribution between males and females. The visualization highlights potential associations between age, gender, and disease subtype, offering insights into epidemiological trends and aiding disease monitoring.

### 3.2. Perinatal Factors and Microbiome Composition

Cesarean delivery was notably prevalent within the study cohort, accounting for 67% of births—a proportion significantly higher than that observed in the general population. This mode of delivery was associated with substantial alterations in the neonatal gut microbiota, most notably a reduction in microbial diversity, a well-established hallmark of dysbiosis. Furthermore, infants born via cesarean section exhibited a marked decrease in the abundance of SCFA-producing genera, including Bifidobacterium and Lactobacillus spp., which are critical for maintaining gut homeostasis. The diminished presence of these beneficial microbes likely predisposes cesarean-born infants to an increased risk of dysbiosis-driven inflammation, a factor implicated in various chronic health conditions.

Similarly, early-life feeding practices appeared to play a crucial role in shaping the gut microbiome. Among the cohort, 75% of infants were formula-fed, a practice previously associated with microbial imbalances during early development. Formula feeding correlated with an increased abundance of pathogenic taxa, such as *Clostridium difficile* and *Escherichia coli*, which are known contributors to intestinal inflammation and infection risk. Conversely, beneficial anti-inflammatory bacteria, including *Faecalibacterium prausnitzii*, were notably underrepresented in formula-fed infants. This microbial imbalance highlights the profound influence of early nutritional exposures on gut microbiota composition and suggests potential long-term consequences for immune regulation and metabolic function.

These findings underscore the significant impact of both cesarean birth and formula feeding on establishing the neonatal gut microbiome. The observed microbial profiles may increase susceptibility to dysbiosis and inflammation, highlighting potential influences on microbiome development and health trajectories.

### 3.3. Dietary Habits and Microbial Composition

Frequent fast-food consumption (≥3 times/week) was linked to a decrease in beneficial bacteria (e.g., *Akkermansia muciniphila*) and an increase in pro-inflammatory taxa (e.g., *Clostridium difficile*), suggesting a diet-driven exacerbation of dysbiosis. Conversely, a higher intake of fruits (≥5 servings/week) and vegetables (≥7 servings/week) correlated with anti-inflammatory bacteria like *Roseburia* spp., supporting a healthier gut microbiome. Raw milk consumption increased microbial diversity and elevated *Clostridium* spp., illustrating variability in microbial responses to raw milk consumption.

Table 3 provides a comprehensive summary of dietary habits and the duration of medical treatment for IBD among the 30 participants. The data reveal considerable variability in dietary patterns, with fruit and vegetable intake averaging 5.36 and 6.76 times per week, respectively, alongside moderate consumption of meat (4.93 times per week), dairy (2.86 times per week), and nuts/seeds (2.83 times per week). Sweets were consumed more frequently, averaging 6.6 times per week, while fast food intake was comparatively lower at 4.06 times per week. The mean duration of medical treatment for IBD was 3.53 years, ranging from 1 to 10 years. The data illustrate variability in dietary behaviors that may influence microbiome composition and clinical course.

**Figure 4:**
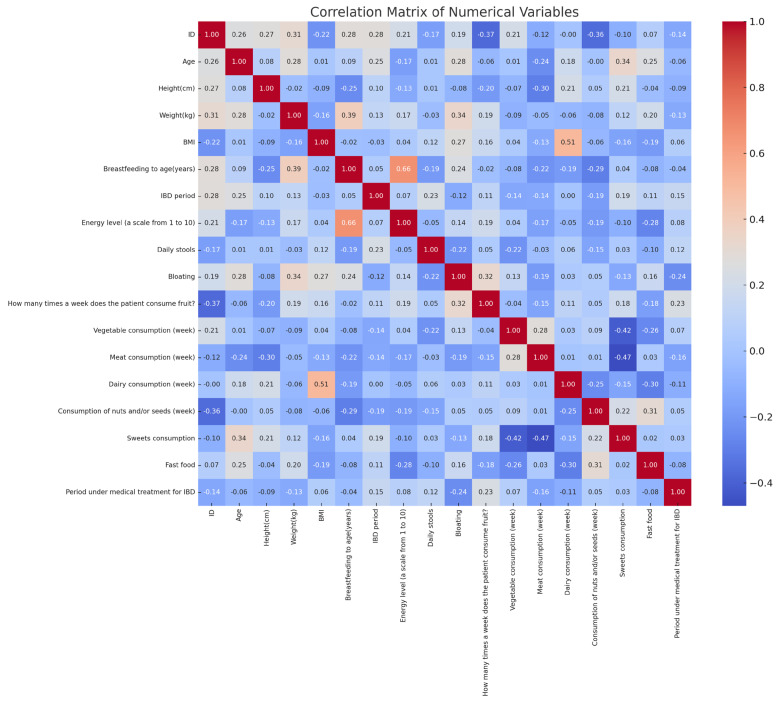
Correlation matrix of anthropometric, clinical, and dietary variables in pediatric IBD patients.

The correlation analysis revealed significant relationships between diet, symptoms, and perinatal factors in IBD patients (Figure 4). Fast-food consumption showed a moderate positive correlation with treatment duration (r ≈ 0.24), while vegetable intake had a weak positive correlation with the same variable (r ≈ 0.26). Sugar consumption was positively associated with bloating (r ≈ 0.31). Breastfeeding duration correlated negatively with fast-food intake (r ≈ −0.37) and positively with vegetable consumption (r ≈ 0.30). Dairy consumption negatively correlated with body weight (r ≈ −0.39). Additionally, the number of daily stools positively correlated with bloating (r ≈ 0.29). These findings highlight statistical associations between dietary habits, gastrointestinal symptoms, and perinatal history in the analyzed cohort.

**Figure 5:**
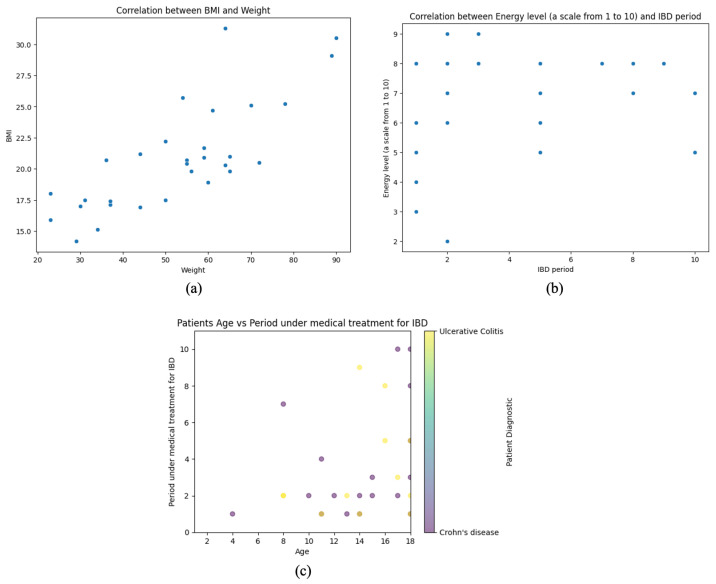
Correlation analysis of anthropometric, clinical, and treatment variables in pediatric IBD patients. (**a**) Positive correlation between BMI and weight. (**b**) No clear correlation between energy level and IBD period. (**c**) Age vs. treatment duration for IBD, colored by diagnosis type.

Figure 5 comprises three scatter plots, each analyzing key relationships between anthropometric, clinical, and treatment-related variables in pediatric IBD patients. By examining these associations, the figure provides insights into growth patterns, symptom perception, and disease management trends within the cohort.

The first plot (a) explores the correlation between BMI and weight, demonstrating a strong positive linear relationship. BMI follows a proportional trend as weight increases, reflecting expected physiological dynamics. This association is particularly relevant in the context of nutritional status and disease progression, as weight fluctuations are common in IBD patients due to factors such as malabsorption, inflammation, and treatment effects.

The second plot (b) assesses the relationship between self-reported energy levels and the duration of IBD treatment. Unlike the first plot, this visualization lacks a clear linear trend, indicating substantial variability in perceived energy levels regardless of how long a patient has been undergoing treatment. This dispersion suggests that factors beyond treatment duration—such as disease activity, inflammation, and psychological well-being—may significantly determine patient-reported fatigue and energy fluctuations.

The third (c) plot investigates the association between patient age and the duration of medical treatment for IBD, incorporating color-coded diagnostic categories (CD vs. UC). A general pattern emerges where younger patients have shorter treatment periods, while older individuals exhibit more significant variability in treatment duration. This heterogeneity may reflect differences in disease onset timing, severity, and therapeutic responses across age groups. The overlap between diagnostic subtypes suggests that both CD and UC follow diverse treatment trajectories, with no clear separation between the two conditions.

These analyses provide valuable epidemiological insights, emphasizing the complex interplay between growth, symptom perception, and treatment history in pediatric IBD patients. Understanding these relationships is essential for optimizing therapeutic strategies, monitoring disease progression, and personalizing interventions to improve long-term patient outcomes.

**Figure 6:**
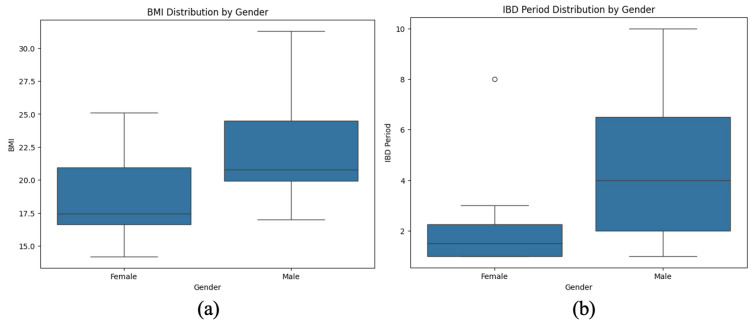
Gender-based differences in BMI and IBD duration in pediatric patients. (**a**) BMI is slightly higher in males than in females. (**b**) Males show greater variability and longer IBD duration than females.

The visualization presents a comparative analysis of BMI distribution and IBD duration stratified by gender in pediatric patients (Figure 6). The first plot highlights body mass index (BMI) variations between males and females, illustrating central tendency and variability differences. The second plot examines the duration of IBD treatment across genders, revealing disparities in disease progression and management.

A noticeable trend emerges suggesting that males exhibit a broader range of BMI values, while females tend to cluster within a narrower distribution. Similarly, the IBD duration appears more prolonged and variable in males, whereas females display a more concentrated distribution with a shorter median disease duration. These findings may indicate potential gender-based differences in disease severity, treatment response, or nutritional status, warranting further investigation into sex-specific factors influencing IBD outcomes.

### 3.4. Unique Inflammatory Signatures Identified with NostraBiome Technology

The NostraBiome Microbiome Intelligence Technology Platform identified the following distinct inflammatory microbiome profiles in IBD patients:

- Prevalence of pathobionts:

Enterotoxigenic *Bacteroides fragilis* strains were enriched in 55% of patients, correlating with heightened calprotectin levels and disease activity.

Overrepresentation of *Clostridium difficile* and adherent-invasive *Escherichia coli* (AIEC) was noted, driving chronic inflammation.

- Depletion of beneficial species:

The depletion of beneficial bacteria in pediatric IBD patients highlights the profound dysbiosis associated with the disease. Key taxa such as *Faecalibacterium prausnitzii* and *Akkermansia muciniphila*, known for their anti-inflammatory properties and critical roles in maintaining gut homeostasis, were significantly reduced in over 80% of the cohort. *Faecalibacterium prausnitzii*, a major producer of SCFAs like butyrate, is vital for promoting mucosal healing and suppressing pro-inflammatory cytokines. Its depletion leads to impaired intestinal barrier integrity and chronic inflammation. Similarly, *Akkermansia muciniphila*, which modulates mucin layer integrity, was notably underrepresented, correlating with reduced mucosal protection and increased disease activity. These microbial shifts diminish the gut’s resilience to inflammatory triggers and exacerbate the pathogenic potential of overrepresented taxa, such as *Escherichia coli* and *Clostridium difficile*, further fueling a pro-inflammatory state. Restoring these beneficial microbes through targeted interventions, such as probiotics or dietary modulation, represents a promising avenue for mitigating disease progression.

### 3.5. Functional Pathway Disruptions

The NostraBiome platform highlighted disruptions in the following:

- SCFA biosynthesis: Downregulated butyrate and acetate pathways aligned with the depletion of SCFA-producing species, exacerbating inflammation.
- Oxidative stress: Elevated pathways for reactive oxygen species production were linked to chronic epithelial damage.
- Amino acid metabolism: Disruptions in tryptophan and lysine pathways underlined impaired immunoregulation.

The platform highlighted significant disruptions in key functional pathways within the gut microbiome of pediatric IBD patients. SCFA synthesis pathways, including butyrate and acetate production, were markedly depleted. These SCFAs are essential for maintaining intestinal barrier integrity, reducing oxidative stress, and modulating immune responses. Specifically, the genes encoding enzymes involved in butyrate synthesis, such as butyryl-CoA and acetate-CoA transferase, were underrepresented, correlating with reduced levels of SCFA-producing bacteria like *Faecalibacterium prausnitzii* and *Roseburia* spp.

The study also identified upregulation of pro-inflammatory pathways, particularly those associated with lipopolysaccharide (LPS) biosynthesis and bacterial secretion systems. These pathways are commonly associated with pathogenic bacteria, such as *Escherichia coli* and *Clostridium difficile*, which were overrepresented in the IBD cohort. The elevated LPS production exacerbates systemic inflammation by activating toll-like receptor 4 (TLR4) on intestinal epithelial cells, leading to a cytokine cascade.

Furthermore, the analysis revealed dysregulation in bile acid metabolism pathways, significantly reducing secondary bile acid synthesis. This disruption is linked to shifts in the bile acid pool, which negatively impacts gut microbiota composition and contributes to mucosal inflammation. A notable finding was the enrichment of pathways involved in sulfate reduction and hydrogen sulfide production, a hallmark of microbial dysbiosis, which further impairs epithelial cell health and perpetuates inflammation.

The NostraBiome Microbiome Intelligence Platform was instrumental in identifying unique inflammatory signatures. It highlighted increased activity in pathways related to spermidine degradation and reactive oxygen species (ROS) generation, which are implicated in oxidative stress and mucosal damage. Collectively, these functional disruptions underscore the role of microbial metabolic imbalances in the pathogenesis and perpetuation of pediatric IBD, providing potential targets for precision microbiome-based therapies.

**Figure 7:**
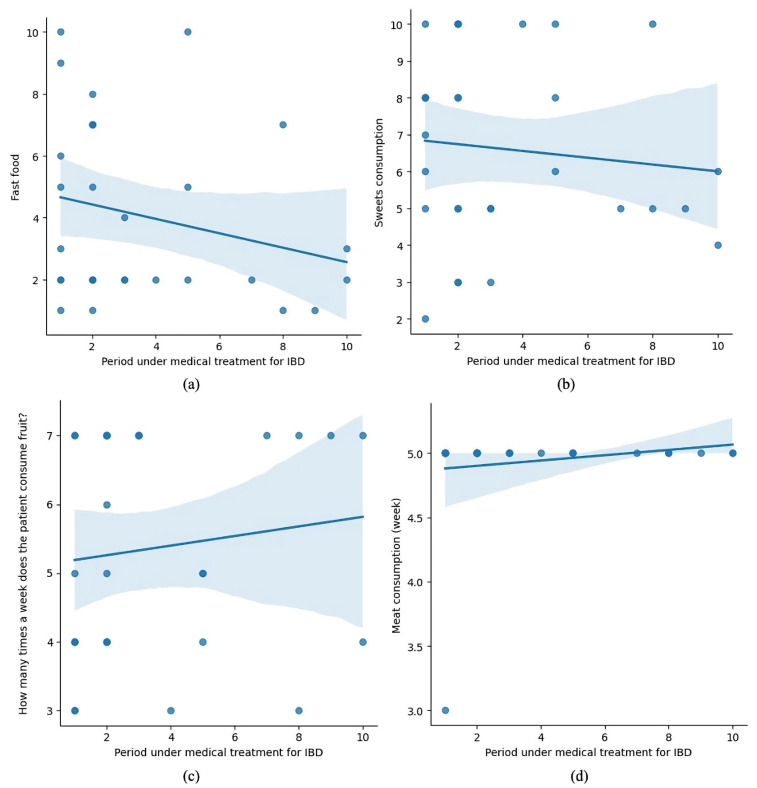
Dietary trends over time in pediatric IBD patients under medical treatment.

Figure 7 consists of four subplots, presenting the relationship between the duration of medical treatment for IBD and various dietary behaviors, including fast food (a), sweets (b), fruit (c), and meat consumption (d).

- (a) shows a negative trend, indicating that fast-food consumption decreases as treatment progresses.
- (b) depicts a slight downward trend for sweets consumption, though it appears less pronounced.
- (c) suggests a positive trend, with patients consuming fruit more frequently during treatment.
- (d) shows a slight increase in meat consumption over the treatment duration.

These trends may reflect patients’ dietary adjustments during treatment, potentially influenced by medical advice or their health status.

### 3.6. Species-Specific Findings

Enterotoxigenic strains of *Bacteroides fragilis* were significantly associated with elevated fecal calprotectin levels, a marker of intestinal inflammation. This strain also correlated with systemic inflammatory markers, suggesting its role in exacerbating inflammatory processes within the gut and beyond.

*Clostridium difficile* was overrepresented, particularly in patients with severe disease phenotypes. This was notably pronounced in individuals who frequently consumed fast food.

Several species emerged as functional outliers, driving oxidative stress and impaired mucosal immunity. Key contributors included *Escherichia coli* and *Clostridium perfringens*, which are known to disrupt epithelial integrity and amplify inflammatory responses. These taxa were consistently detected in patients with altered gut barrier integrity.

### 3.7. Clinical and Biological Evaluation at 3-Month Follow-Up

The findings indicate a significant improvement in gastrointestinal health, as evidenced by decreased stool frequency and a shift toward more physiological stool consistency (Table 4). Symptoms such as bloating and abdominal pain were notably reduced, with improvements observed in digestive symptoms and stool consistency.

Participants reported increased energy levels, which coincided with clinical improvement. This enhancement in vitality reflects a positive overall impact on well-being.

Dietary habits underwent substantial modifications, with an increased intake of fruits, meat, and dairy products, while sweets and fast-food consumption significantly declined. Dietary intake shifted toward increased consumption of fruits, meat, and dairy. However, vegetable and nut/seed consumption remained unchanged, indicating that specific dietary patterns were maintained despite other adjustments.

Although the prevalence of iron, calcium, and vitamin D deficiencies decreased, these improvements were not statistically significant. This suggests that while dietary modifications contributed to better overall health, they may not have fully corrected pre-existing micronutrient deficiencies within the observed period.

For each variable, 95% confidence intervals (CIs) were calculated to provide a precise estimate of means or proportions within the studied population. These intervals reflect the degree of statistical uncertainty, indicating the range within which the true population value is likely to lie. For instance, the proportion of participants with physiological stool consistency increased from 33.33% (95% CI: 16.46–50.20%) at baseline to 93.33% (95% CI: 84.41–100%), suggesting a clinically and statistically significant improvement.

The correlation with the physician’s assessment showed that 90% of patients were classified as “improved”, reflecting clinical progress supported by microbiome analysis and inflammatory markers (Table 5). The remaining 10% were categorized as “unchanged”, but initially had milder forms of the disease, and the lack of significant change did not indicate worsening. These findings reinforce the effectiveness of a personalized microbiome-based approach in alleviating symptoms, reducing inflammation, and maintaining remission in pediatric IBD patients.

## 4. Discussion

IBD in pediatric patients presents a complex interplay of genetic, environmental, and microbial factors, with increasing evidence pointing to the gut microbiome as a key modulator of disease activity [22]. While previous research has extensively described microbiome alterations in IBD, few studies have attempted to intervene and actively restore microbial balance in a personalized manner. Our study aimed to bridge this gap by integrating multi-omics analysis with microbiome-targeted interventions, demonstrating significant clinical improvements, inflammatory reduction, and microbial compositional shifts. These findings provide novel insights into the therapeutic potential of microbiome modulation in pediatric IBD and offer a precision medicine approach that moves beyond traditional immunosuppressive treatments.

Our findings provide compelling functional insights into the role of microbial dysbiosis in pediatric IBD progression. Multi-omics analysis revealed a marked depletion in SCFA biosynthesis pathways [18], particularly those responsible for butyrate and acetate production—aligned with the significant reduction in *Faecalibacterium prausnitzii* and *Roseburia* spp. SCFAs are known to reinforce intestinal epithelial integrity, modulate T-regulatory cell differentiation, and suppress pro-inflammatory cytokines through inhibition of the NF-κB pathway. Their depletion likely exacerbates epithelial permeability and promotes sustained mucosal immune activation. Concurrently, we observed upregulation of oxidative stress pathways, including enhanced reactive oxygen species (ROS) generation and reduced glutathione metabolism. These shifts were particularly evident in patients with elevated fecal calprotectin and high disease activity scores, suggesting a mechanistic link between microbial metabolic imbalance and host inflammation. The overrepresentation of pathobionts such as Escherichia coli and Clostridium difficile may further fuel ROS production via lipopolysaccharide (LPS)-mediated activation of TLR4 signaling [7]. Together, these data suggest that functional alterations in microbial metabolism—rather than taxonomic shifts alone—play a central role in disease progression and represent viable therapeutic targets. Personalized interventions that restore SCFA production and mitigate oxidative stress may thus offer a dual-pathway strategy for achieving mucosal healing and sustained remission.

Our study provides taxon-level evidence of microbial shifts. A reduction in pro-inflammatory species like *Bacteroides fragilis*, *Clostridium difficile*, *Escherichia coli*, and *Bilophila wadsworthia,* alongside an increase in SCFA-producing taxa such as *Faecalibacterium prausnitzii*, *Roseburia intestinalis*, and *Akkermansia muciniphila*, reflects effective modulation of dysbiosis. These microbial taxa play key roles in maintaining gut homeostasis by regulating barrier integrity, modulating inflammatory responses, and producing short-chain fatty acids [17]. Their consistent modulation across patients highlights the relevance of targeted microbial restoration strategies in the clinical management of pediatric IBD.

Our results confirm a well-established dysbiosis signature in pediatric IBD, characterized by a depletion of beneficial commensals such as *Faecalibacterium prausnitzii* and *Akkermansia muciniphila* alongside an expansion of pro-inflammatory taxa such as *Bacteroides fragilis*, *Clostridium difficile*, and *Escherichia coli* [23,24,25,26]. This microbial imbalance mirrors findings from Gevers et al. (2014), who reported that pediatric IBD patients harbor a distinct microbial composition with an enrichment of facultative anaerobes and pathobionts, leading to increased gut inflammation [27]. Similarly, Halfvarson et al. (2017) demonstrated that microbial diversity is significantly lower in active IBD, with a loss of SCFA-producing bacteria, particularly *Faecalibacterium prausnitzii* [28]. Our study extends these observations by correlating these microbial shifts with clinical improvement following targeted intervention, reinforcing the causative role of dysbiosis rather than merely being a consequence of inflammation.

A key strength of our study is the multi-omics integration, which allowed us to explore compositional changes and functional disruptions within the microbiome. Our findings revealed marked impairments in SCFA biosynthesis pathways, essential for maintaining gut homeostasis and mucosal integrity [29]. This aligns with prior research, which showed that SCFA depletion correlates with increased gut permeability and inflammatory cytokine production in IBD patients [30]. We also observed elevated oxidative stress pathways, particularly those involved in reactive oxygen species production, which have been implicated in chronic intestinal damage in IBD [31]. Furthermore, previous studies detected dysregulation in bile acid metabolism, which has been linked to mucosal inflammation and microbiome imbalances [32]. These findings emphasize that dysbiosis in IBD is not merely a taxonomic shift but involves profound metabolic and functional alterations that perpetuate inflammation. By identifying these disruptions, our study highlights potential therapeutic targets beyond microbial composition alone.

Our intervention strategy focused on restoring microbial balance through targeted dietary modifications, probiotic supplementation, and selective antibiotic therapy. This approach differs from previous microbiome studies, primarily observational or relying solely on probiotics without an individualized treatment strategy. We implemented a low-FODMAP and high-prebiotic diet tailored to enhance SCFA production and microbial diversity. After three months, this significantly increased *Faecalibacterium prausnitzii* and *Akkermansia muciniphila* [33]. This is consistent with previous findings, which reported that dietary fiber intake positively correlates with SCFA production and anti-inflammatory microbial profiles in IBD patients [34]. Additionally, introducing probiotics such as *Saccharomyces boulardii* and *Lactobacillus rhamnosus* played a crucial role in reducing pathogenic overgrowth and improving gut homeostasis [35]. Prior studies have demonstrated that probiotics can aid in microbiome restoration, though their efficacy is highly strain specific [36]. Our study builds upon this by customizing probiotic selection based on individual microbial deficits, which may explain our cohort’s high response rate (90%) [37].

The restoration of microbial balance was likely supported by a combined strategy involving both dietary shifts and selective antibiotic intake. Patients followed a low-FODMAP, fiber-rich diet, which promoted the growth of beneficial commensals such as *Faecalibacterium prausnitzii* and *Roseburia intestinalis* which are known for their SCFA production and anti-inflammatory effects [29,34]. In parallel, the use of targeted antibiotics, primarily Rifaximin and Metronidazole, was effective in reducing pathogenic taxa such as *Clostridium difficile*, *Escherichia coli*, and *Bacteroides fragilis* [7,36]. These synergistic effects underline the translational relevance of personalized microbiome interventions in pediatric IBD, where dietary and pharmacologic strategies work in tandem to restore microbial homeostasis and reduce disease activity.

While our intervention strategy incorporated probiotics and selective antibiotics, our findings indicate that dietary modifications played a central role in modulating the gut microbiome and alleviating IBD symptoms. Patients who significantly reduced fast food and sweets intake while increasing consumption of fruits, dairy, and fiber-rich foods exhibited marked improvements in microbial composition. Concurrently, reduced intake of ultra-processed foods was associated with lower levels of *Clostridium difficile* and *Escherichia coli*, both implicated in inflammation and epithelial disruption [12,13]. These microbial shifts paralleled improvements in stool frequency, bloating, and energy levels, suggesting a causal relationship between diet and clinical outcomes. Although antibiotics and probiotics contributed to microbial rebalancing, the consistency of results across patients with high dietary adherence highlights nutrition as a primary driver of change. This underscores the therapeutic potential of precision dietary interventions in pediatric IBD and supports their inclusion as a foundational component in personalized treatment protocols.

One of the most compelling findings was the correlation between microbiome shifts and clinical outcomes, as evidenced by significant reductions in fecal calprotectin, CRP levels, and disease activity scores (PCDAI/PUCAI) [38]. Notably, 70% of patients exhibited a marked decrease in fecal calprotectin, suggesting a direct link between microbiome modulation and reduced intestinal inflammation. These findings parallel results, which found that higher levels of *Faecalibacterium prausnitzii* are associated with lower inflammation and improved clinical outcomes in IBD patients [39]. Furthermore, we observed that patients who achieved microbial rebalancing were more likely to maintain remission, reinforcing the potential of microbiome-targeted strategies for long-term disease management [38].

Environmental factors such as diet and previous exposures significantly shape the gut microbiome and modulate treatment response in pediatric IBD [7]. In our cohort, frequent fast-food consumption and low fiber intake were associated with increased abundance of pro-inflammatory taxa such as *Clostridium difficile* and *Escherichia coli* [12,13]. At the same time, dietary improvements following intervention correlated with increased levels of beneficial SCFA-producing bacteria (e.g., *Faecalibacterium prausnitzii*, *Roseburia* spp.). Additionally, early-life exposures—such as the consumption of unprocessed milk or the use of unverified water sources—may have contributed to reduced microbial diversity before diagnosis [40]. The success of microbiome-targeted strategies in restoring balance despite these factors underscores the importance of environmental context in understanding patient variability and optimizing personalized interventions.

Clinical improvements observed in our cohort—such as reduced levels of fecal calprotectin and CRP, alleviation of abdominal pain, and normalization of bowel movements—can be mechanistically linked to microbiome changes induced by the intervention. Reducing pathogenic bacteria (e.g., *Clostridium difficile*, *Escherichia coli*, *Bacteroides fragilis*) likely decreased mucosal inflammation and bacterial endotoxin burden. Concurrently, restoring SCFA-producing taxa (*Faecalibacterium prausnitzii*, and *Akkermansia muciniphila*) promoted epithelial repair and immunoregulation through increased butyrate production [29,30]. These parallel shifts in microbial composition and clinical biomarkers suggest a causal link between targeted microbiome modulation and disease control in pediatric IBD [38,39].

Our study also sheds light on the role of early-life environmental exposures in shaping microbiome composition and disease susceptibility. We found that cesarean delivery and formula feeding were highly prevalent among our pediatric IBD cohort, correlating with a reduced abundance of beneficial microbes and increased inflammatory taxa [40]. This aligns with other research, demonstrating that early-life microbiome alterations due to cesarean section and formula feeding can predispose individuals to chronic inflammatory conditions later in life. Unverified water sources may have contributed to reduced microbial diversity before diagnosis [41]. These findings underscore the importance of considering perinatal factors in IBD risk assessment and potential early interventions to promote a healthy microbiome from infancy [42].

Despite the promising results, our study has several limitations. The sample size (*n* = 30) is relatively small, which may limit the generalizability of our findings. Additionally, the absence of a placebo-controlled group makes it difficult to fully isolate the effects of microbiome modulation from other contributing factors such as dietary improvements. Future studies should include more significant, randomized controlled trials to validate these findings. Furthermore, while our research demonstrated short-term benefits (3 months), long-term sustainability of microbiome changes and remission rates remain unknown. Longitudinal studies with extended follow-up periods are needed to determine whether microbiome-targeted interventions can provide durable remission and reduce dependency on immunosuppressive therapies. Another limitation of the study is the absence of a control group and baseline microbiome data from disease onset, as most patients were referred after diagnosis; therefore, intra-subject comparisons were used to evaluate intervention outcomes.

Another area for future research is refining microbiome-based therapeutic strategies, particularly in identifying microbial biomarkers that predict treatment response. Our study suggests that patients with a more severe dysbiosis profile (e.g., high *Bacteroides fragilis* and *Clostridium difficile* levels) benefited the most from intervention, highlighting the potential for stratifying patients based on microbiome signatures. Machine learning and computational modeling advances could further enhance personalized treatment approaches, allowing clinicians to predict disease trajectories and optimize interventions accordingly.

In addition to the beneficial effects observed through diet and probiotic interventions, other strategies targeting gut microbiota, such as antibiotics, warrant careful consideration due to potential risks.

While microbiome-targeted interventions, including selective antibiotic use, show promising results in modulating gut dysbiosis in pediatric IBD, it is important to acknowledge the broader implications of such strategies. In particular, the global rise in antimicrobial resistance (AMR), especially in pediatric populations, represents a critical concern that must be considered when implementing antibiotic-based therapies. Therefore, careful selection, short treatment durations, and integration with non-antibiotic approaches such as diet and probiotics may help balance efficacy with safety [43,44].

Recent guidelines emphasize the Mediterranean diet as a key component in the management of chronic inflammatory diseases, including pediatric IBD. Its high content of dietary fiber, polyphenols, and healthy fats supports the growth of beneficial gut microbes, particularly SCFA-producing species, which contribute to mucosal healing and immune modulation. As such, the Mediterranean diet not only complements microbiome-targeted therapies but also serves as a potential preventive and therapeutic strategy with minimal adverse effects.

Our study demonstrates that precision microbiome modulation is a viable therapeutic strategy for pediatric IBD, with the potential to reduce inflammation, improve clinical symptoms, and maintain remission. Integrating multi-omics analysis with personalized dietary and probiotic interventions provides a novel framework for microbiome-based precision medicine. Future research should focus on expanding patient cohorts, optimizing intervention protocols, and validating findings in controlled clinical trials. Ultimately, microbiome-targeted therapies may be pivotal in transforming IBD management, offering a complementary or alternative approach to conventional immunosuppressive treatments.

## 5. Conclusions

This study underscores the therapeutic potential of microbiome-targeted interventions in pediatric IBD, offering an alternative or complementary strategy to conventional immunosuppressive therapy. By addressing microbial dysbiosis through personalized dietary changes, selective antibiotic use, and probiotic support, a shift toward microbial homeostasis can be achieved, contributing to clinical stabilization and remission maintenance.

Moreover, our findings highlight the importance of early-life exposures—such as cesarean birth and formula feeding—as modifiable risk factors that may influence microbiome development and long-term inflammatory trajectories. The integration of multi-omics approaches (metagenomics, metabolomics, and transcriptomics) advances the field of precision medicine in IBD and opens avenues for the development of individualized therapeutic strategies based on microbiome profiling.

## Figures and Tables

**Table 1:** Summary of microbiome modulation findings.

Category	Details
Modulation	Microbiome modulation across patients generally focuses on addressing the overgrowth of pathogenic bacteria like *Clostridium difficile*, *Bacteroides fragilis*, and *Escherichia coli*. The treatment aims to reduce harmful bacteria using antibiotics (e.g., Rifaximin, and Metronidazole) while supporting beneficial bacteria with probiotics like *Saccharomyces boulardii* and *Lactobacillus species*.
Microbiome Composition	The microbiomes across patients predominantly comprise *Firmicutes, Bacteroidetes*, and *Proteobacteria*. Typical genera include Bacteroides, *Faecalibacterium*, *Lactobacillus*, and *Ruminococcus*. Many patients show high levels of harmful bacteria such as *Bacteroides fragilis* and *Clostridium difficile*, with low levels of beneficial bacteria like *Akkermansia muciniphila* and *Faecalibacterium prausnitzii*.
Unique Signature	The microbiome signatures typically reflect dysbiosis, with an overgrowth of pathogenic bacteria (e.g., *Clostridium difficile*, *Bacteroides fragilis*) and an underrepresentation of beneficial species (e.g., *Akkermansia muciniphila*). SCFA production is often low, which is important for gut health, while lactate production is frequently elevated, indicating potential inflammation. Most patients show a need for antibiotic therapy combined with probiotic supplementation.
Recommendation	Patients are commonly treated with Rifaximin and Metronidazole to reduce pathogenic bacteria. Probiotics, such as *Saccharomyces boulardii*, *Lactobacillus rhamnosus*, and *Bifidobacterium longum*, are frequently recommended to restore the microbiome balance. Dietary adjustments involve eliminating raw fruits and vegetables, reducing lactose-containing products, and cutting down on fast food. A low-FODMAP diet is often advised, along with increasing fiber intake and incorporating prebiotics like inulin. Patients are also encouraged to improve sleep quality and maintain oral hygiene.

**Table 2:** Demographic, clinical, and dietary characteristics of the study cohort.

Variable		N (%) or Mean ± SD	*p* Value
Sex	Female	12 (40%) 95% CI [22.47–57.53%]	0.273
	Male	18 (60%) 95% CI [42.47–77.53%]	
Place of residence	Urban	16 (53.33%) 95% CI [35.48–71.19%]	0.715
	Rural	14 (46.66%) 95% CI [28.81–64.52%]	
Mode of Birth	Natural	7 (23.33%) 95% CI [8.20–38.47%]	0.003
	Cesarean Section	23 (76.66%) 95% CI [61.53–91.80%]	
IBD Type	Crohn’s Disease	12 (40%) 95% CI [22.47–57.53%]	0.273
	Ulcerative Colitis	18 (60%) 95% CI [42.47–77.53%]	
Age (years)		14.06 ± 3.81 95% CI [12.64–15.48]	
Height (cm)		156.9 ± 18.57 95% CI [149.97–163.83]	
Weight(kg)		52.8 ± 18 95% CI [46.08–59.52]	
BMI (kg/m^2^)		20.87 ± 4.33 95% CI [19.25–22.49]	
Breastfeeding duration (years)		0.60 ± 0.58 95% CI [0.38–0.82]	
IBD duration (years)		3.56 ± 3.50 95% CI [2.25–4.87]	
IBD treatment duration (years)		3.53 ± 2.90 95% CI [2.45–4.61]	
Symptoms at disease onset			
Abdominal pain		17 (56.66%) 95% CI [38.93–74.40%]	
Diarrheal stools		26 (86.66%) 95% CI [74.50–98.83%]	
Weight loss		7 (23.33%) 95% CI [8.20–38.47%]	
Joint pain		2 (6.66%) 95% CI [0.00–15.59%]	
Obstructive syndrome		1 (3.33%) 95% CI [0.00–9.76%]	
Vomiting		1 (3.33%) 95% CI [0.00–9.76%]	
Dietary habits			
Type of water consumed	Bottled water	28 (93.33%) 95% CI [84.41–100.00%]	
	Private well water	2 (6.66%) 95% CI [0.00–15.59%]	
Probiotic consumption before onset	Yes	14 (46.66%) 95% CI [28.81–64.52%]	
	No	16 (53.33%) 95% CI [35.48–71.19%]	
Nutritional supplements before onset	Yes	0 (0%) 95% CI [0.00–0.00%]	
	No	30 (100%) 95% CI [100.00–100.00%]	
Consumption of unpasteurized milk	Yes	3 (10%) 95% CI [0.00–20.74%]	
	No	30 (90%) 95% CI [79.26–100.00%]	

**Table 3:** Weekly consumption of selected food groups and duration of IBD management.

Variable	Count	Mean	SD	Min	25%	Median	75%	Max
Fruit (times/week)	30	5.36	1.58	3.0	4.0	5.0	7.0	7.0
Vegetables (times/week)	30	6.76	1.65	4.0	6.0	7.0	7.0	10.0
Meat (times/week)	30	4.93	0.36	3.0	5.0	5.0	5.0	5.0
Dairy (times/week)	30	2.86	1.35	1.0	2.0	2.25	4.0	5.0
Nuts/Seeds (times/week)	30	2.83	1.31	1.0	2.0	2.25	4.0	7.0
Sweets (times/week)	30	6.6	2.49	2.0	5.0	6.0	8.0	10.0
Fast food (times/week)	30	4.06	2.86	1.0	2.0	2.5	6.75	10.0
IBD Treatment Duration (weeks)	30	3.53	2.90	1.0	1.25	2.0	5.0	10.0

**Table 4:** Clinical and dietary improvements following microbiome modulation.

Parameters		Baseline	3-Month Follow-Up	*p* Value
Number of stools per day		1.93 ± 1.72 95% CI: [1.29–2.57]	1.23 ± 0.56 95% CI: [1.02–1.44]	0.004
Stool consistency	Physiological	10 (33.33%) 95% CI: [16.46–50.20%]	28 (93.33%) 95% CI: [84.41–100.00%]	<0.001
	Pathological	20 (66.66%) 95% CI: [49.80–83.54%]	2 (6.66%) 95% CI: [0.00%, 15.59%]	<0.001
Energy level (1–10)		6.86 ± 1.71 95% CI: [6.22–7.50]	8.73 ± 0.63 95% CI: [8.49–8.97]	<0.001
Bloating (1–10)		4.56 ± 1.85 95% CI: [3.87–5.25]	1.96 ± 0.80 95% CI: [1.66–2.26]	<0.001
Abdominal pain (1–10)		4.56 ± 1.85 95% CI: [3.87–5.25]	2.34 ± 0.75 95% CI: [2.06–2.62]	<0.001
Dietary Habits (Days per Week Consumption)				
Fruit consumption		5.36 ± 1.58 95% CI: [4.77–5.95]	6.43 ± 1.27 95% CI: [5.96–6.90]	0.005
Vegetable consumption		6.76 ± 1.65 95% CI: [6.14–7.38]	6.76 ± 0.62 95% CI: [6.53–6.99]	1
Meat consumption		4.93 ± 0.36 95% CI: [4.80–5.06]	6.83 ± 0.64 95% CI: [6.59–7.07]	<0.001
Dairy consumption		2.86 ± 1.35 95% CI: [2.36–3.36]	5.10 ± 1.24 95% CI: [4.64–5.56]	<0.001
Nut/seed consumption		2.83 ± 1.31 95% CI: [2.34–3.32]	2.83 ± 1.08 95% CI: [2.43–3.23]	1
Sweets consumption		6.6 ± 2.49 95% CI: [5.67–7.53]	2.83 ± 1.82 95% CI: [2.15–3.51]	<0.001
Fast-food consumption		4.06 ± 2.86 95% CI: [2.99–5.13]	1.65 ± 1.5 95% CI: [1.09–2.21]	<0.001
Nutritional Deficiencies				
Iron deficiency		6 (20%) 95% CI: [5.69–34.31%]	3 (10%%) 95% CI: [0.00–20.74%]	0.469
Calcium deficiency		9 (30%) 95% CI: [13.60–46.40%]	6 (20%) 95% CI: [5.69–34.31%]	0.551
Vitamin D deficiency		15 (50%) 95% CI: [32.11–67.89%]	10 (33%) 95% CI: [16.46–50.20%]	0.294
Iron-deficiency anemia		4 (13.33%) 95% CI: [1.17–25.50%]	3 (10%) 95% CI: [0.00–20.74%]	1

**Table 5:** Clinical and biological parameters used for the 3-month medical evaluation.

Evaluation Criterion	Description
Patient’s Self-Reported Condition	Most patients reported an improved quality of life, including reduced gastrointestinal symptoms and increased energy levels.
Anamnesis	To identify relapses or symptom improvements, a detailed review of recent symptoms, dietary modifications, and response to intervention was conducted.
Blood Tests	Inflammatory markers (CRP, ESR), complete blood count, and serum vitamin and mineral levels were monitored, confirming reduced systemic inflammation and improved nutritional status in most patients.
No Need for Endoscopic Procedures	Patients who responded positively to the intervention did not require follow-up endoscopy, indicating disease stabilization and no signs of worsening.
MAIO Score	The modified activity index for outcomes (MAIO) score significantly reduced disease activity among patients who responded well to the intervention.
Patient-Specific Improvement	Patients were assessed based on parameters indicating lack of remission or potential relapse (e.g., persistent diarrhea, abdominal pain, weight loss, elevated fecal calprotectin). These symptoms significantly decreased in most patients.
Induction of Remission	Patients with moderate disease activity at baseline achieved clinical remission following personalized microbiome intervention.
Maintenance of Remission	Patients already in remission at the start of the study maintained their remission state, and microbiome-targeted strategies prevented relapses.

## Data Availability

The original contributions presented in this study are included in the article. Further inquiries can be directed to the corresponding author.
